# High‐Performance Bio‐Based Epoxy Vitrimers and Composites: Synergizing Robustness and Reprocessability via Fractional Free Volume Regulation

**DOI:** 10.1002/advs.77917

**Published:** 2026-09-27

**Authors:** Kai Dong, Di Zhao, Chengji Zhao

**Affiliations:** ^1^ Key Laboratory of High Performance Plastics Ministry of Education College of Chemistry Jilin University Changchun People's Republic of China

**Keywords:** bio‐based vitrimer, carbon fiber‐reinforced composites, degradation and reprocessability, free volume, self‐healing epoxy resin

## Abstract

The trade‐off between crosslink density and segment mobility challenges bio‐based epoxy vitrimers to simultaneously achieve high mechanical robustness and dynamic adaptability. Here, we report that engineering the fractional free volume serves as an effective structural parameter to decouple this longstanding conflict. By designing bifunctional and trifunctional epoxy monomers from renewable resources, we construct dynamic networks integrating rigid conjugated Schiff bases with flexible siloxane segments, enabling deliberate manipulation of free volume characteristics within the hybrid architecture. Molecular dynamics simulations reveal that the bifunctional system, which possesses a moderately higher fractional free volume, simultaneously achieves high crosslink density and enhanced segmental mobility. This unique combination promotes efficient stress dissipation and accelerates bond exchange kinetics, successfully reconciling strength and toughness without compromising network dynamics. Consequently, the optimized vitrimer exhibits exceptional mechanical properties alongside rapid stress relaxation and self‐healing capability. When employed as a composite matrix, it enables non‐destructive carbon fiber recovery, as well as adhesive‐free welding and thermoforming. This work establishes fractional free volume as a practical design lever for creating high‐performance, sustainable covalent adaptable networks, thereby providing a route from empirical toughening toward topology‐directed property optimization.

## Graphical abstract

In this work, two bio‐based epoxy monomers were designed to tune the dynamic network of a rigid–flexible hybrid epoxy through free‐volume control, achieving a synergy among high free volume, high cross‐linking density, and segmental mobility while balancing strength and toughness.

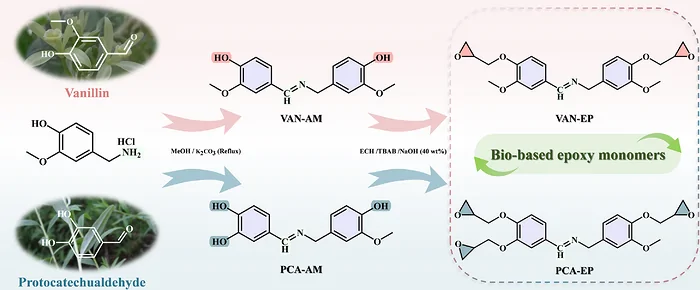

## 1 Introduction

Epoxy resins are among the most widely used thermosetting polymers in industrial applications due to their exceptional mechanical strength, corrosion resistance, as well as chemical and thermal stability. Among commercial epoxy resins, bisphenol A diglycidyl ether (DGEBA), derived from petroleum‐based bisphenol A (BPA), holds the largest market share. The polar functional groups (e.g., hydroxyl and epoxy groups) in these resins enable the fabrication of lightweight, high‐strength, and high‐modulus carbon fiber‐reinforced composites (CFRCs) through versatile processing techniques. These composites are employed in critical industries such as aerospace, wind energy, and specialized equipment manufacturing [1, 2, 3, 4]. However, the permanent covalent crosslinked networks of DGEBA‐based epoxy systems intrinsically prevent reprocessing and recycling, resulting in severe sustainability challenges and growing environmental concerns [5, 6, 7].

To address these limitations, bio‐based epoxy resins containing covalent adaptive networks, particularly epoxy vitrimers, have emerged as promising alternatives. By incorporating dynamic covalent bonds such as disulfides [8, 9], Schiff bases [10, 11], β‐hydroxy esters [12], or siloxane linkages [13], vitrimer systems enable network rearrangement under external stimuli, thus imparting reprocessability, self‐healing, chemical degradation, and recyclability [14]. For instance, Zhang et al. reported a novel eugenol‐derived epoxy vitrimer through a bio‐based polymerization approach involving reversible transesterification reaction [15]. It can be readily reprocessed by a simple chemical decomposition in a benign ethanol solution without the addition of extra catalysts. Wang's group developed a fully bio‐based recyclable epoxy vitrimer by constructing a rigid‐flexible dynamic covalent network from renewable resources, featuring a rigid conjugated structure derived from ferulic acid and a flexible fatty acid backbone with dynamic disulfide groups [1, 16]. Through the cleavage of disulfide bonds and hydrolysis of ester bonds, both the resin matrix and carbon fibers from its CFRCs can be completely recovered. Despite significant advances in developing various epoxy vitrimers from biomass feedstocks, the performance of currently reported bio‐based epoxy resins has yet to fully surpass that of petroleum‐based counterparts. The inherent trade‐off between strength, toughness, and network dynamics in epoxy resins remains a fundamental challenge [10, 17, 18, 19].

High crosslinking densities are typically required to achieve superior mechanical stiffness and strength, yet they severely restrict segmental mobility, leading to brittleness and sluggish bond exchange kinetics. Conversely, reducing crosslink density enhances chain mobility and dynamic responsiveness, but often at the expense of mechanical robustness. Extensive efforts have been devoted to overcoming this dilemma through approaches such as rubber toughening, thermoplastic blending, incorporation of inorganic nanofillers, and construction of hierarchical cross‐linking networks [20, 21, 22]. However, these strategies often rely on compositional complexity or extrinsic additives, which can disrupt network homogeneity, impair recyclability and reprocessability, and introduce phase separation or compatibility issues. Achieving a synergistic balance between mechanical strength, toughness, and dynamic adaptability in additive‐free, single‐component vitrimers has rarely been systematically exploited [23].

In this work, we present a free‐volume engineering strategy to prepare high‐performance bio‐based epoxy resins by constructing rigid‐flexible dynamic crosslinked networks containing rigid conjugated Schiff base scaffolds and flexible siloxane segments. This is realized by the rational design of bifunctional and trifunctional epoxy monomers (VAN‐EP and PCA‐EP) derived from renewable resources, followed by curing with 1,3‐bis(3‐aminopropyl) tetramethyldisiloxane (BAS). Rigid‐flexible dynamic cross‐linked networks are capable of deliberately tuning the size and spatial distribution of free volume, while ensuring high cross‐linking density and excellent segment mobility within the epoxy resin network. From a molecular engineering perspective, free volume plays a crucial role in balanced rigid‐flexible cross‐linked networks through a bifunctional mechanism. First, acting as a viscoelastic “shock absorber,” it furnishes nanoscale voids that accommodate conformational relaxation of rigid segments, enhance segmental mobility, dissipate energy, and relieve local stress concentrations. Second, serving as a molecular “lubricant,” it boosts segmental dynamics and markedly accelerates network rearrangement under thermal stimuli. This synergy enables bio‐based epoxy resins to achieve an optimal balance among mechanical strength, toughness, and reprocessability. Notably, the bifunctional epoxy vitrimer VAN‐BAS exhibits a rare combination of high tensile strength (94.3 MPa), excellent toughness (impact strength 39.1 kJ·m^−2^), rapid stress relaxation, and high recyclability, owing to its moderate free volume and crosslink density. Furthermore, when used as a matrix for CFRCs, this material enables non‐destructive carbon fiber recovery through solvent‐induced degradation and supports adhesive‐free welding and thermoforming. This work demonstrates that tuning the size and spatial distribution of free volume simultaneously regulates segmental relaxation behavior and bond exchange kinetics without sacrificing mechanical robustness, providing a promising strategy for obtaining high‐performance, sustainable epoxy vitrimers and their CFRCs.

## 2 Results and Discussion

### 2.1 Design of Bio‐Based Epoxy Vitrimers Incorporating Schiff Base Structures

**FIGURE 1:**
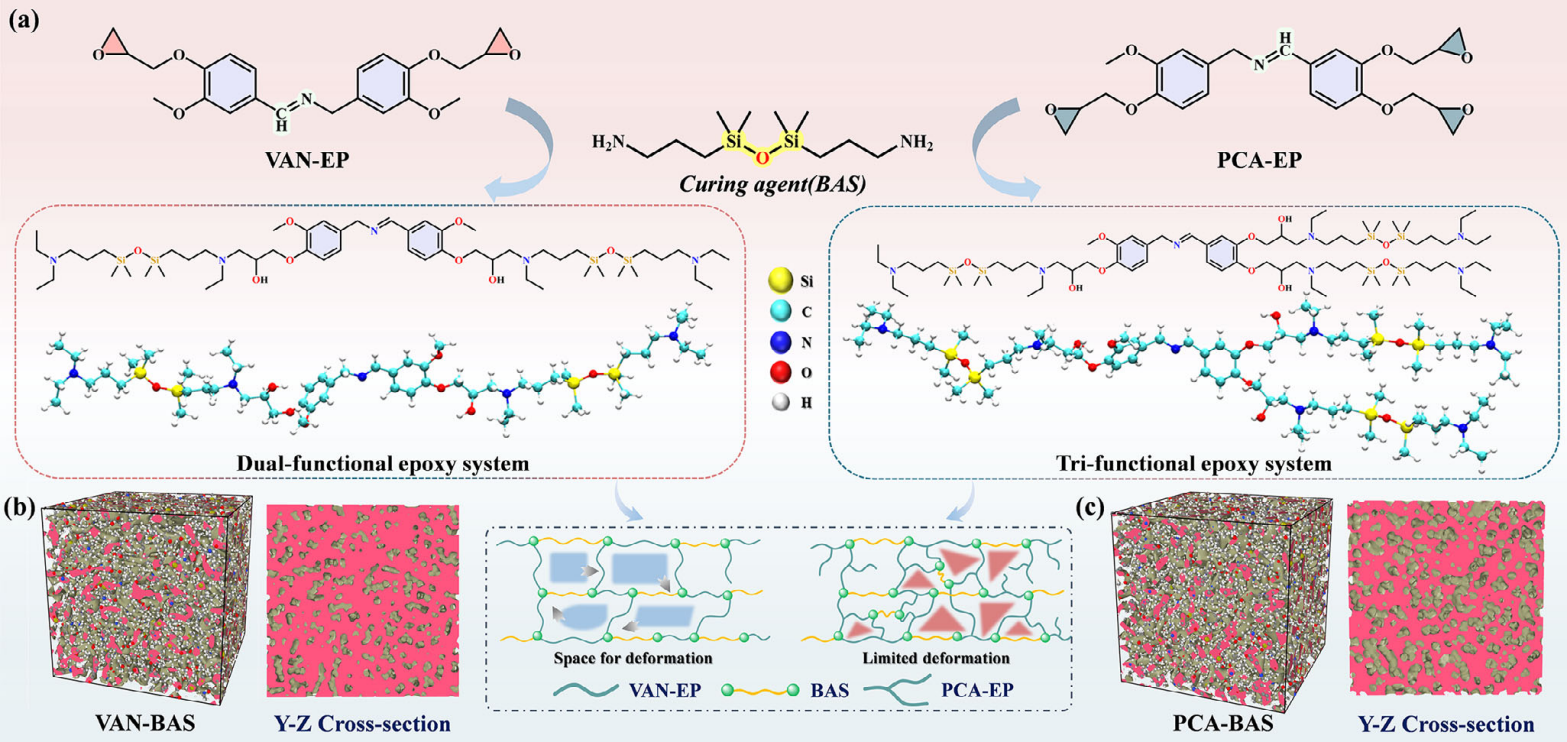
(a) Schematic illustration of the bio‐based epoxy vitrimer structure and its crosslinked network. Molecular structures and free volume distributions of (b) VAN‐BAS and (c) PCA‐BAS, where the brown surfaces correspond to the occupied volume and the pink regions represent the free volume.

Incorporating dynamic cross‐linked networks imparts reprocessability, self‐healing capacity, and recyclability to traditional thermosetting epoxy resins. However, these dynamic covalent bonds often compromise mechanical strength and thermal stability, thus restricting their practical utility [11]. Achieving a simultaneous optimization of mechanical robustness and dynamic adaptability in epoxy vitrimers fundamentally requires appropriate control over network topology. In this work, we propose fractional free volume as a governing structural descriptor that bridges crosslink density, chain mobility, and macroscopic performance. To validate this concept, two bio‐based epoxy monomers with distinct functionalities were rationally designed: a bifunctional vanillin‐derived monomer (VAN‐EP) and a trifunctional protocatechualdehyde‐derived monomer (PCA‐EP). Upon curing the resulting monomers with a flexible siloxane‐containing amine (BAS), rigid‐flexible crosslinked networks featuring dual dynamic covalent bonds were formed (Figure 1a). The varied functionalities of epoxy monomers inherently alter the crosslink density and topological constraints of the resulting epoxy vitrimers. Meanwhile, this design balances the rigidity of the cross‐linked network with the flexibility of the polymer chains, thereby synergistically optimizing the dynamic exchange behavior and mechanical properties [17, 18, 24].

**SCHEME 1:**
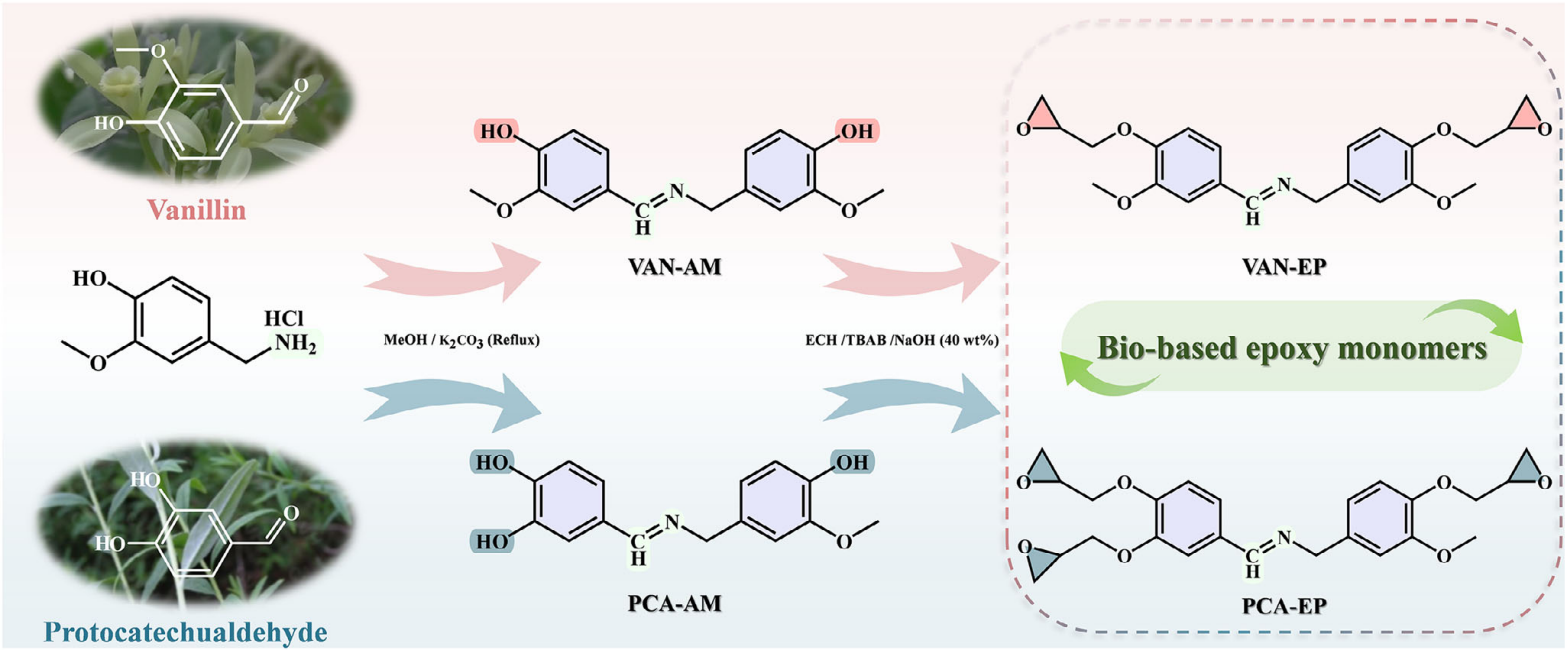
Synthetic route of bifunctional and trifunctional bio‐based epoxy monomers.

The synthetic route of these bio‐based epoxy monomers is illustrated in Scheme 1. The chemical structures of VAN‐EP and PCA‐EP were confirmed by their ^1^H NMR and Fourier transform infrared (FT‐IR) spectroscopy. As shown in Figure S1, the FT‐IR spectra of the bio‐based bisphenol monomers exhibited characteristic absorption peaks corresponding to the imine bond (1640 cm^−1^) and hydroxyl group (∼ 3200 cm^−1^). High‐resolution mass spectrometry (HRMS; Figures S2 and S3) further confirmed that the measured molecular weights were consistent with the calculated theoretical values of the bisphenol monomers. The Schiff base moiety introduced through amine‐aldehyde condensation remained stable during the subsequent epoxidation of the phenolic hydroxyl groups. In the ^1^H NMR spectra (Figure S4), the proton corresponding to the ─C═N group remained unchanged at 8.2–8.3 ppm, whereas the phenolic hydroxyl signal at 9.1–9.2 ppm disappeared and was replaced by characteristic epoxy proton signals. The epoxy proton signals of the bifunctional epoxy monomers resonated at 2.7–2.8 and 3.3 ppm, whereas those of the trifunctional analogs were observed in the ranges of 2.7–2.9, 3.3–3.6, and 4.1–4.4 ppm [11]. Furthermore, the appearance of the characteristic epoxy absorption band at 910 cm^−1^ in Figure S5 confirmed the successful synthesis of the target epoxy monomers.

The disappearance of the characteristic epoxy absorption bands and the emergence of broad hydroxyl bands in Figure S5 confirmed that the epoxy‐amine curing reaction was completed. To gain deeper insight into the network topology of the cured vitrimers, molecular dynamics simulations were employed to probe the internal network structure and examine the spatial distribution and conformational behavior of the molecular chains within the crosslinked networks. As shown in Figure 1b, the bifunctional system (VAN‐BAS) exhibited a significantly high fractional free volume of 38.3%, which is 29% higher than that of the trifunctional PCA‐BAS system (29.7%). Furthermore, as shown in Figure 1c, the VAN‐BAS system has a larger free volume than the PCA‐BAS system [19]. The reduced fractional free volume of the PCA‐BAS system can be attributed to the excessive crosslinking points within the trifunctional crosslinked networks, leading to dense chain packing and localized confinement of the chain segments.

**FIGURE 2:**
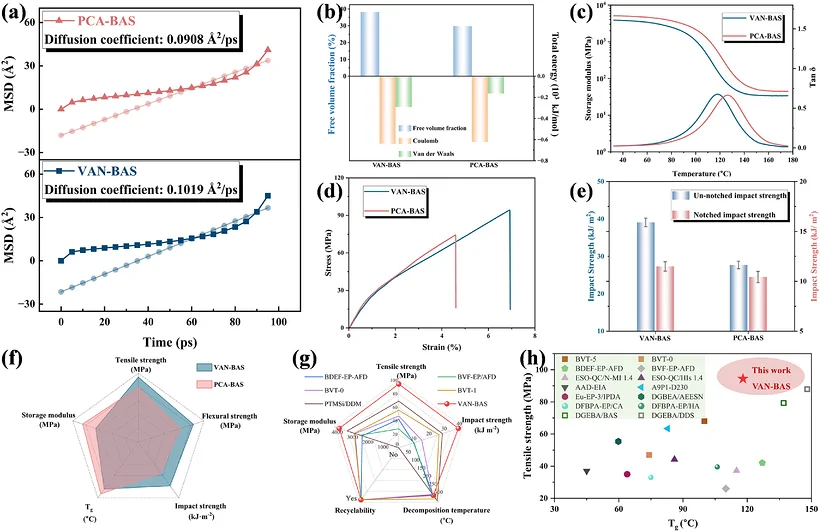
(a) Mean square displacement (MSD) by molecular dynamics simulations, (b–e) calculated free volume, total energy, and mechanical properties (including tensile strength, storage modulus, T_g_, and impact strength) of VAN‐BAS and PCA‐BAS. Comprehensive performance comparison of (f) VAN‐BAS and PCA‐BAS, (g, h) VAN‐BAS and previously reported epoxy vitrimers.

Additionally, molecular dynamics simulations were performed to calculate the diffusion coefficients of both cured vitrimers, which serve as a measure of translational molecular mobility (Figure 2a). The diffusion coefficient of VAN‐BAS (0.1019 Å^2^·ps^−1^) is 12% higher than that of PCA‐BAS (0.0908 Å^2^·ps^−1^). According to free volume theory, the reduced diffusion coefficient of PCA‐BAS originates from a molecular‐structure‐induced increase in the critical volume, which prevents molecules accessing sufficient free volume for molecular motion during free‐volume fluctuations. Consequently, the trifunctional crosslinked system with a smaller fractional free volume is constrained by the rigidity arising from excessive crosslinking, resulting in markedly reduced molecular mobility [25]. Although the Coulombic interactions of both vitrimers are comparable, the van der Waals energies of VAN‐BAS and PCA‐BAS are −290 and −163 kJ·mol^−^^1^, respectively (Figure 2b). The more negative van der Waals energy of the VAN‐BAS system indicates that a moderate free volume facilitates more efficient chain arrangement and stabilizes intermolecular interactions. These results suggest that free volume is not merely a passive consequence of crosslinking, but rather a tunable structural parameter governing network topology and intermolecular organization. Importantly, a moderately distributed free volume enables the network to maintain structural integrity while ensuring adequate mobility of the molecular segments and providing sufficient configurational space for segmental rearrangement.

### 2.2 Excellent Mechanical Robustness and Durability Via Regulation of Free Volume

The effect of free volume on the mechanical performance of epoxy vitrimers was subsequently investigated by dynamic mechanical analysis (DMA) and stress–strain curves. As shown in Figure 2c, PCA‐BAS with a lower free volume fraction exhibited a higher storage modulus (5115 MPa at 30°C) and glass transition temperature (127°C) than those of VAN‐BAS (3900 MPa at 30°C and 116°C, respectively). These results can be attributed to the higher crosslink density of PCA‐BAS. According to rubber elasticity theory, the crosslink density of PCA‐BAS was calculated to be 3880 mol·m^−3^, 41% higher than that of VAN‐BAS (Table S1). However, the limited segmental mobility within this rigid network resulted in brittle mechanical behavior. Detailed mechanical performance data for the bio‐based epoxy resins are summarized in Table S2. VAN‐BAS exhibited a tensile strength of 94.3 MPa, a flexural strength of 168.7 MPa, and an impact strength of 39.1 kJ·m^−^^2^, surpassing those of PCA‐BAS by 26%, 31%, and 46%, respectively (Figure 2c–e, Figures S6 and S7). In contrast, PCA‐BAS displayed a Young's modulus of 1.8 GPa and a flexural modulus of 5.3 GPa, which were 27% and 26% higher than those of VAN‐BAS, respectively, indicating its greater stiffness. Consequently, the high stiffness of PCA‐BAS resulted in increased brittleness, rendering it susceptible to brittle fracture and poor crack resistance [11]. Conversely, VAN‐BAS utilized a free‐volume‐mediated stress dissipation mechanism, maintaining an epoxy network with an appropriate crosslinking density and superior overall mechanical properties that uniquely combine high strength and toughness (Figure 2f).

Specifically, the moderately distributed free volume in VAN‐BAS serves dual roles. First, it provides nanoscale‘buffer zones'that accommodate local chain deformation, thereby mitigating stress concentration. Second, it enhances segmental mobility, enabling efficient energy dissipation during crack propagation [2, 26]. Fracture surface analysis further supported this interpretation. VAN‐BAS exhibited a rough, fish‐scale‐like morphology with abundant fine wrinkles, indicative of significant plastic deformation, whereas PCA‐BAS displayed a smooth and flat river‐pattern morphology, characteristic of a typical brittle fracture mode (Figure S8). These findings demonstrate that free volume enables the decoupling of strength and toughness in crosslinked epoxy networks, thereby overcoming the conventional trade‐off associated with high crosslink density. Compared with previously reported bio‐based epoxy vitrimers, VAN‐BAS demonstrated superior overall mechanical properties, including a tensile strength exceeding 90 MPa and an impact strength approaching 40 kJ·mol^−2^. Moreover, it exhibited a storage modulus of 3.9 GPa and a thermal decomposition temperature of 300°C. Crucially, it can be degraded and recycled through subsequent processing, making it highly suitable as a resin matrix for CFRCs (Figure 2g). Furthermore, the VAN‐BAS prepared herein showed higher tensile strength and glass transition temperature than those of recently reported epoxy vitrimers, highlighting its strong potential for improving mechanical and thermomechanical performance through tailored structural design. Highly cross‐linked epoxy resins generally exhibit pronounced rigidity, affording excellent thermal stability but substantial brittleness. By balancing high crosslink density with mechanical toughness, VAN‐BAS achieved a competitive synergy between thermal and mechanical properties by tuning the free volume to optimize the rigid‐flexible network (Figure 2h and Table S3). Bio‐based VAN‐BAS is not a universal substitute for all petroleum‐based epoxy resins; however, it is a highly competitive alternative for applications that require high strength, toughness, and sustainability.

**FIGURE 3:**
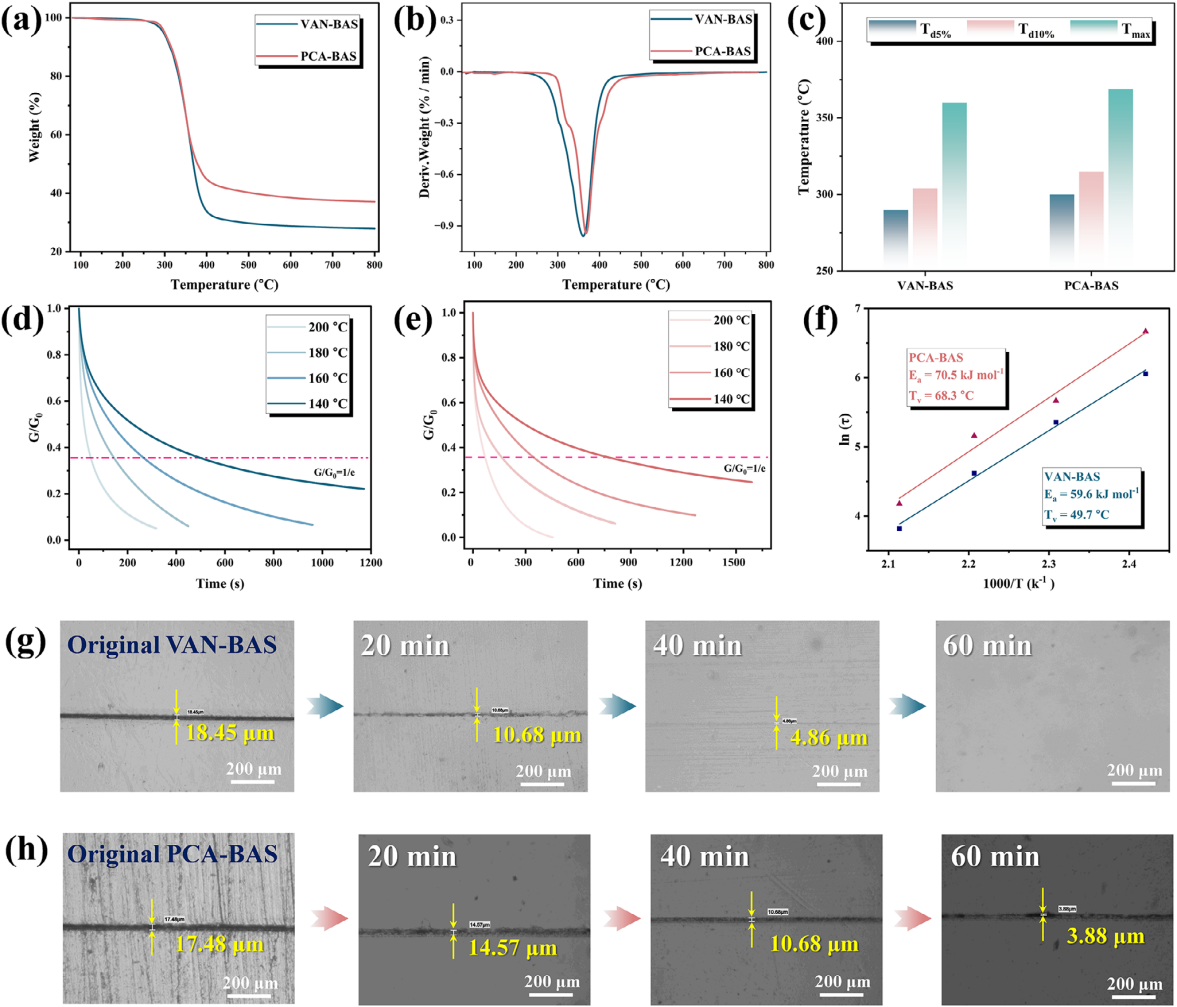
TGA (a) and DTG (b) curves, and the corresponding thermal decomposition parameters (c) of VAN‐BAS and PCA‐BAS. Normalized shear stress relaxation curves of VAN‐BAS (d) and PCA‐BAS (e). (f) Arrhenius plots derived from the measured relaxation times (τ^∗^) for the epoxy vitrimers. Self‐healing performance of VAN‐BAS (g) and PCA‐BAS (h) at 200°C.

The thermal stability of the bio‐based epoxy vitrimers was evaluated using thermogravimetric analysis (TGA), and the results are presented in Figure 3a and Table S4. The thermal decomposition temperatures (*T*_d5%_) of VAN‐BAS and PCA‐BAS were 294°C and 307°C, respectively (a difference of 13°C), while their maximum decomposition temperatures (*T*_max_) were 360°C and 369°C, respectively (Figure 3b,c). Despite the significant difference in crosslink density, the variation in thermal degradation resistance was relatively small. This behavior was attributed to the strong intermolecular interactions arising from the conjugated imine bonds, which suppressed molecular thermal motion and delayed thermal decomposition [27]. PCA‐BAS exhibited a denser crosslinked network owing to its higher crosslink density, which significantly restricted polymer chain mobility. At elevated temperatures, this restricted mobility promoted the formation of a highly stable crosslinked char residue. The resulting carbonaceous layer acted as a thermal barrier, protecting the underlying material from further decomposition and thereby resulting in a higher char yield at 800°C [28].

### 2.3 Fast Self‐Healing Vitrimers via Dual Dynamic Bonds

Stress relaxation analysis was conducted to investigate the network rearrangement kinetics in crosslinked systems featuring dual dynamic exchange mechanisms [8, 9, 29, 30]. The activation energy (*E*_a_) for the topological rearrangement was calculated using the Arrhenius equation, and the detailed parameters are provided in Tables S5 and S6. As illustrated in Figure 3d,e, increasing the temperature from 140°C to 200°C reduced the relaxation time (τ*) of VAN‐BAS from 426 to 45 s and that of PCA‐BAS from 786 to 65 s, confirming the significantly faster relaxation kinetics of VAN‐BAS. Therefore, VAN‐BAS showed a lower activation energy (59.6 kJ·mol^−1^) compared to PCA‐BAS (70.5 kJ·mol^−1^). Accordingly, the topological freezing transition temperatures (T_v_) of VAN‐BAS and PCA‐BAS, as extrapolated from the Arrhenius plots, were determined to be 49.7°C and 68.3°C, respectively (Figure 3f). This difference can be attributed to the interplay between free volume and bond exchange dynamics. In the densely crosslinked PCA‐BAS network, restricted segmental motion imposes steric constraints on bond exchange reactions, thereby increasing the energy barrier for network rearrangement [31, 32]. Although PCA‐BAS contains a significantly higher total content of dynamic covalent bonds than VAN‐BAS, it exhibits slower stress relaxation kinetics and a higher activation energy. This counterintuitive result indicates that the concentration of dynamic covalent bonds alone does not determine the kinetics of network reorganization. Instead, the dense cross‐linking of the PCA‐BAS network imposes topological constraints that severely restrict segmental mobility, as evidenced by its lower fractional free volume and diffusion coefficient. These steric hindrances raise the energy barrier for bond exchange and thus outweigh the potential kinetic benefits of the higher dynamic covalent bond density. By contrast, a moderate cross‐linking density and a larger free volume provide sufficient configurational freedom for bond reorganization, leading to faster stress relaxation and a lower activation energy [12, 33, 34, 35].

To evaluate the self‐healing performance of the bio‐based epoxy vitrimers, surface scratches were generated using a surgical blade and subsequently thermally activated at 200°C to trigger Schiff base and siloxane bond exchange [13, 36]. The evolution of scratch width was quantified at 20 min intervals using polarized light microscopy, and the corresponding data are summarized in Table S7. VAN‐BAS exhibited superior self‐healing efficiency, achieving nearly complete scratch closure within 60 min, whereas PCA‐BAS showed significantly slower healing kinetics (Figure 3g,h). Despite identical thermal treatment, the scratch width of PCA‐BAS decreased by only 16% and 38% after 20 and 40 min, respectively. Even after 60 min, the scratch remained visible despite a 77% reduction in width. These results confirm that free volume plays a critical role in governing the dynamic bond exchange kinetics of vitrimer systems.

**FIGURE 4:**
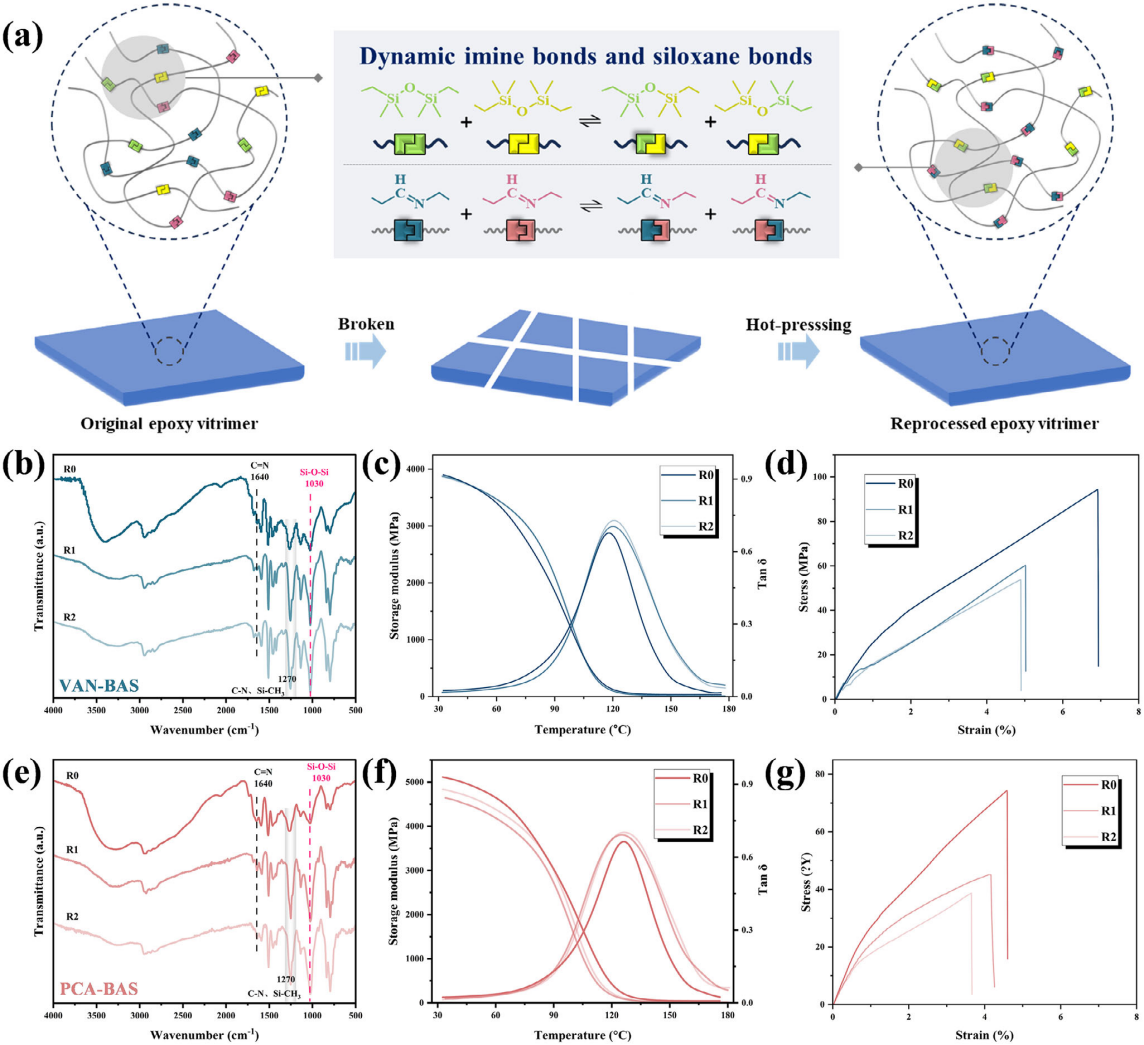
(a) Schematic illustration of the recycling process of epoxy vitrimers and the exchange mechanisms of dynamic siloxane and imine bonds. (b) FT‐IR spectra, (c) DMA thermograms, and (d) stress–strain curves of pristine and reprocessed VAN‐BAS; (e) FT‐IR spectra, (f) DMA thermograms, and (g) stress–strain curves of pristine and reprocessed PCA‐BAS.

The reprocessability of the dynamic crosslinked networks was evaluated by pulverizing the specimens into powder and subsequently hot‐pressing at 200°C and 15 MPa for 30 min. The reshaped epoxy resin exhibited a smooth, grain‐free surface (Figure S9). As illustrated schematically in Figure 4a, the polymer network underwent topological rearrangement driven by dynamic covalent bond exchange under thermal stimulation. This bond exchange also relaxed internal residual stresses, thereby mitigating the performance degradation typically associated with thermal aging. As shown in Figure 4b,e, the characteristic absorption bands of C═N, Si─O─Si, and ─OH were retained in the reshaped epoxy vitrimers, demonstrating the preservation of their structural integrity during the reshaping process [37, 38, 39].

DMA measurements confirmed that the reprocessed materials exhibited thermomechanical properties comparable to those of the pristine samples (Table S8). The dynamic nature of the epoxy vitrimer networks enabled efficient reprocessing via hot pressing. For VAN‐BAS, this additional cross‐linking increased T_g_ to 120°C after reprocessing, while the storage modulus remained stable (Figure 4c). The slight increase in glass transition temperature after reprocessing suggested that residual curing reactions and network rearrangement contributed to structural densification. In contrast, despite a slight decrease in the storage modulus of PCA‐BAS, its crosslink density remained high, and the *T*_g_ retained essentially unchanged (Figure 4f). Although multiple reprocessing cycles are theoretically possible, the cumulative effects of structural defects and thermal aging during repeated hot‐pressing must be considered [40].

Specifically, the tensile strength of VAN‐BAS decreased to 60 MPa (64% retention) after the first cycle and further to 53 MPa (57% retention) after the second cycle (Figure 4d). For comparison, the tensile strength of PCA‐BAS decreased to 45 MPa (61% retention) and 38 MPa (50% retention) after the first and second cycles, respectively (Figure 4g). It can be observed that the performance degradation of PCA‐BAS after thermal remolding was more pronounced for PCA‐BAS than VAN‐BAS, highlighting the importance of network flexibility enabled by free volume. Sufficient segmental mobility allowed effective interfacial fusion and defect healing during the reprocessing of VAN‐BAS. Conversely, the highly constrained PCA‐BAS network was more susceptible to irreversible damage accumulation and inefficient defect relaxation. Reprocessed bio‐based epoxy resins can exhibit substantial losses in mechanical strength. However, future research is expected to improve the mechanical property retention of recycled materials through strategies such as optimizing reprocessing conditions, introducing additional dynamic crosslinking mechanisms, or adding nanofillers.

### 2.4 Applications of CFRCs With Both Reprocessability and Recyclability

**FIGURE 5:**
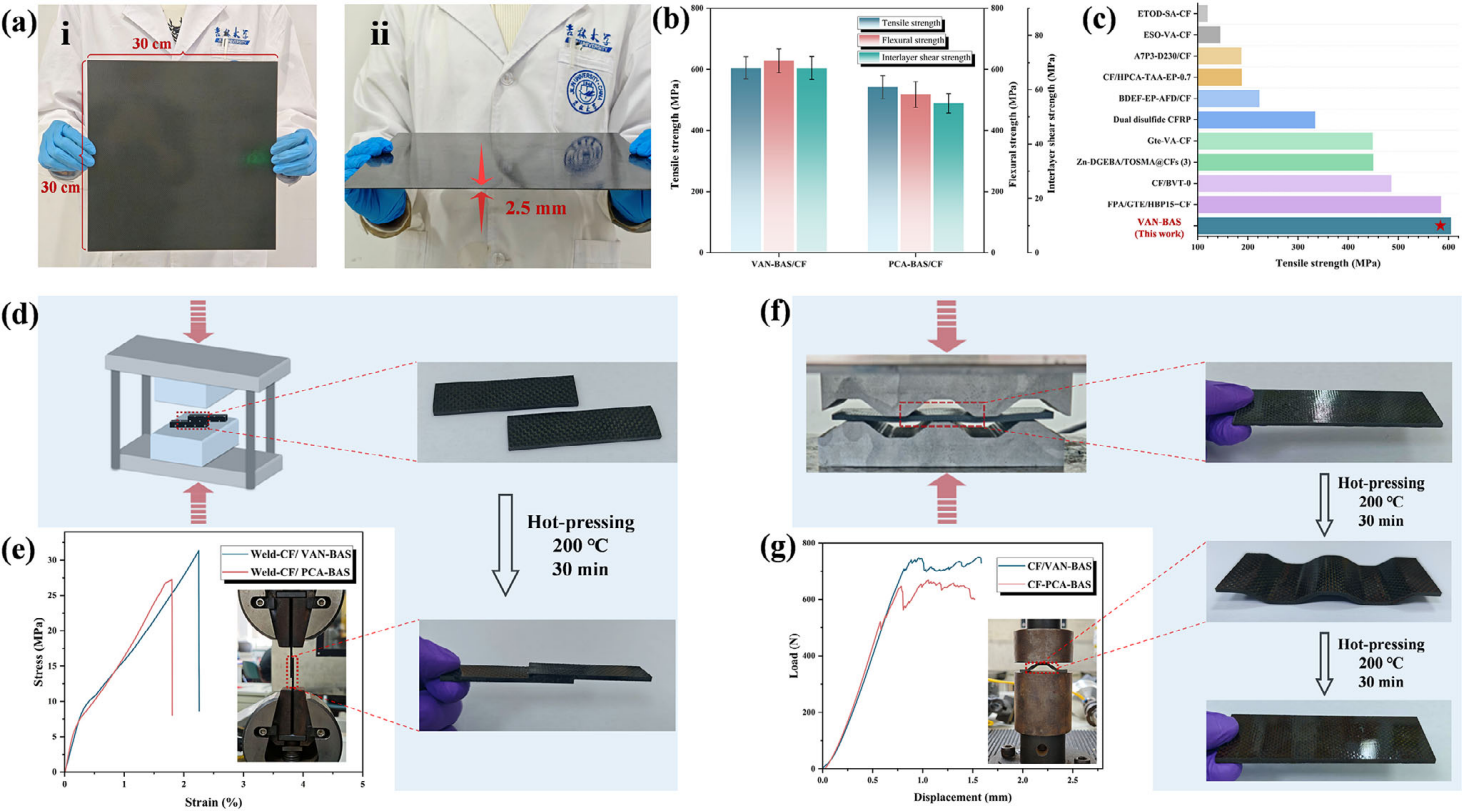
(a) Digital photograph of the bio‐based epoxy composite. (b) Mechanical properties of CF/VAN‐BAS and CF/PCA‐BAS. (c) Performance comparison between CF/VAN‐BAS and previously reported bio‐based composites containing dynamic covalent bonds. (d, f) Demonstrations of the dynamic reprocessing and thermoforming of the bio‐based epoxy composites. (e) Lap shear stress–strain curves for the bonded composite laminates. (g) Load‐displacement curves of the reconfigured 3D composite components.

Epoxy resins form crosslinked networks while their epoxy groups undergo ring‐opening reactions during curing to generate abundant hydroxyl groups. This polyhydroxy structure promotes a strong and tough resin‐fiber interface, making epoxy the predominant matrix material for CFRCs [41]. With continuously improving mechanical performance and increasingly advanced structural designs, bio‐based epoxy resins are poised to replace their petroleum‐based counterparts as key materials for high‐performance, multifunctional CFRCs. To further validate the practical applicability of the proposed design, the optimized vitrimer was employed as the matrix for CFRCs. As depicted in Figure 5a, large‐scale CFRC panels (300 mm × 300 mm × 2.5 mm) were successfully fabricated via compression molding, and the thickness of CFRC panels could be tailored by varying the number of carbon fiber plies. As shown in Figure 5b and Table S9, the resulting CF/VAN‐BAS composite possessed a tensile strength of 605 MPa, a flexural strength of 628 MPa, and an interlaminar shear strength (ILSS) of 68 MPa. These values represent increases of 11%, 21%, and 24%, respectively, compared with those of CF/PCA‐BAS. The superior mechanical strength of CF/VAN‐BAS stems from the robust interface between the high‐strength matrix and carbon fibers, as well as from the free‐volume engineering of the crosslinked epoxy network, which decoupled strength and toughness. This decoupling alleviated the stress concentrations inherent to highly crosslinked networks, thereby enabling effective stress transfer and preventing brittle fracture. CF/VAN‐BAS demonstrated substantial advantages over previously reported dynamically crosslinked CFRC systems (Figure 5c and Table S10).

Furthermore, the dynamic network enabled adhesive‐free welding and thermoforming of CFRC structures, which are typically unattainable for conventional thermosets. The ability to reshape and weld composites under thermal stimuli underscores the advantages of integrating dynamic covalent chemistry with free‐volume‐engineered network structures [42]. The overlapping contact area between the laminates was 20 × 10 mm^2^ (Figure 5d). The welded composite successfully sustained a 3 kg load, approximately 1000 times its own weight, demonstrating significant interfacial strength (Figure S10). To quantitatively evaluate the welding performance, lap shear strength (LSS) tests were conducted (Figure 5e). The welded CF/VAN‐BAS and CF/PCA‐BAS composites exhibited LSS values of 31.3 and 27.2 MPa, respectively, confirming their excellent weldability. Welding composites via dynamic covalent bonds is an effective joining technique that can overcome practical limitations such as high mold costs and press‐size restrictions. The literature on vitrimer composite welding was reviewed, and our results were compared with those of previous studies (Table S11). The lap‐shear strength of 31.3 MPa for CF/VAN‐BAS is significantly higher than values reported in earlier studies and is competitive with, or even slightly superior to, recent advanced welding methods (e.g., 25 MPa for surface degradation‐assisted welding and 30 MPa for zero‐adhesive‐layer welding). These results demonstrate that the resin matrix, engineered through free‐volume design, endows the composite with excellent interfacial bond exchange capacity and welding performance. Nevertheless, further improvements in welding performance should be explored, including optimization of welding process parameters, surface treatment and modification, and joint design, to provide valuable references for future research.

CFRC laminates were positioned within a preheated serrated mold and reconfigured using hot‐pressing. Upon cooling to ambient temperature, a stable, W‐shaped 3D composite component was successfully obtained [8]. Utilizing the same hot‐pressing parameters, the W‐shaped 3D composite was restored to its original planar geometry without discernible interlaminar delamination (Figure 5f). The reconfigured components sustained peak compressive loads of 748 N for CF/VAN‐BAS and 667 N for CF/PCA‐BAS, demonstrating superior load‐bearing capacity and structural integrity (Figure 5g). These results demonstrate that free‐volume engineering not only governs molecular‐scale behavior but also translates into macroscopic functionality and engineering applicability, thereby offering a viable pathway toward sustainable high‐performance composites.

**FIGURE 6:**
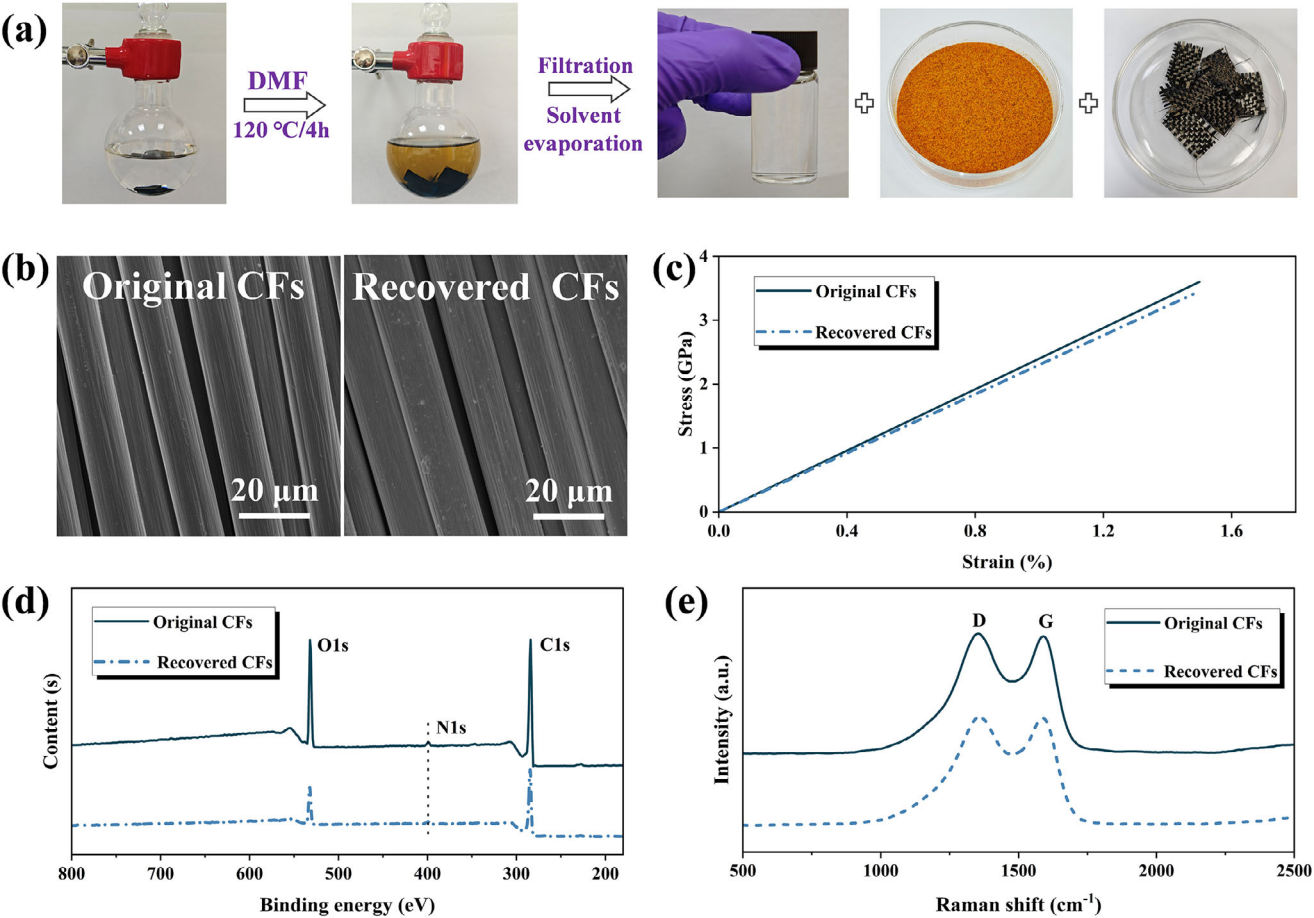
(a) Digital photographs documenting the solvent assisted recovery process of the bio‐based CFRC. Subsequent to the treatment, the reclaimed DMF solvent, fragmented resin particles, and carbon fiber fabric were obtained. (b) SEM images, (c) tensile stress–strain curves, (d) XPS spectra, and (e) Raman spectra of pristine and reclaimed CFs.

Initially, the chemical resistance of the resin matrices was evaluated using a range of common organic solvents. After immersion for 12 h at ambient temperature, the resins exhibited excellent resistance to various solvents (Figure S11). Increasing the temperature to 60°C promoted solvent diffusion, leading to the gradual leaching of residual unreacted species from the crosslinked network. Upon continuous stirring in DMF at 120°C, the crosslinked network underwent significant swelling as evidenced by the progressive opacity and volume expansion of the resin specimens (Figure S11). Within the initial 1–2 h, the resin blocks became highly swollen and softened; with extended stirring (4 h), the swollen networks fragmented into small polymer particles. In contrast, the reference VAN‐CAD resin, which contains no flexible siloxane segments and exhibits limited segmental mobility, showed substantially less swelling under identical conditions. This comparison highlights the beneficial role of tailored free volume in facilitating solvent penetration and network disintegration [12, 43]. Therefore, the tailored free volume facilitated solvent‐induced swelling, enabling non‐destructive matrix degradation and efficient carbon fiber recovery (Figure S12). After the recovered fragments were ground and hot‐pressed into a homogeneous transparent specimen, the FT‐IR absorption peaks remained nearly identical to those of the pristine resin, confirming the excellent chemical stability of epoxy vitrimers throughout the swelling process. The recoverability of CF/VAN‐BAS was evaluated under identical conditions (Figure 6a). After soaking CF/VAN‐BAS in DMF for 4 h, the carbon fibers were fully recovered, while the solvent was recovered via rotary evaporation, with at least 90% of the solvent being reusable. The solvent‐assisted recovery process effectively preserves fiber properties and recovers a significant proportion of the solvent; however, its environmental impact must still be considered. Notably, this process does not require a catalyst. Further optimization will help assess the process's full life cycle environmental impacts and techno‐economic feasibility. SEM images revealed a smooth and defect‐free surface on the recovered carbon fibers (Figure 6b), which retained 96% of their original strength (Figure 6c). XPS and Raman spectroscopy (Figure 6d,e) further demonstrated that the recovered fibers maintained a stable surface elemental composition and a graphitization degree comparable to those of the pristine fibers, confirming the mild and effective nature of the recycling process.

## 3 Conclusions

In summary, we demonstrate that tuning fractional free volume provides a viable strategy to address the intrinsic conflict between mechanical robustness and dynamic adaptability in epoxy vitrimers. Through the molecular design of rigid‐flexible networks derived from renewable resources, we show that a moderate free volume is critical for balancing an appropriate crosslink density with sufficient chain mobility, thus enabling both high strength and toughness performance and rapid bond exchange kinetics. VAN‐BAS achieved a unique combination of high strength and toughness, overcoming the traditional trade‐off associated with highly crosslinked epoxy networks. The dual dynamic crosslinked networks facilitated rapid stress relaxation and enabled adhesive‐free welding and thermoforming of CFRC structures, which are typically unattainable for conventional thermosets. Moreover, the tailored free volume promoted solvent‐induced swelling, allowing non‐destructive matrix degradation and high‐value carbon fiber recovery. This work not only delivers a high‐performance and recyclable bio‐based epoxy platform, but also establishes free‐volume engineering as a broadly applicable design strategy for designing covalently adaptive network polymers with simultaneously enhanced strength, toughness, and dynamic functionality.

## Author Contributions

**Di Zhao**: methodology, software, formal analysis, data curation. **Kai Dong**: conceptualization, investigation, methodology, visualization, writing – original draft, software, formal analysis, data curation. **Chengji Zhao**: funding acquisition, validation, writing – review and editing, project administration, resources, supervision.

## Conflicts of Interest

The authors declare no conflicts of interest.

## Supporting information




**Supporting File**: advs77917‐sup‐0001‐SuppMat.docx.

## Data Availability

The data that support the findings of this study are available on request from the corresponding author. The data are not publicly available due to privacy or ethical restrictions.
