# Supplementary material for: High‐Performance Bio‐Based Epoxy Vitrimers and Composites: Synergizing Robustness and Reprocessability via Fractional Free Volume Regulation
Source: Adv Sci (Weinh). 2026 Sep 27:e77917. Online ahead of print. doi: 10.1002/advs.77917 (PMC13616263; doi:10.1002/advs.77917)
Supplement: Supplementary file 1 [file ADVS-9999-e77917-s001.docx]

**Supporting Information**

**High-Performance Biobased Epoxy Vitrimers and Composites: Synergizing Robustness and Reprocessability via Fractional Free Volume Regulation**

Kai Dong^a^, Di Zhao^a^, Chengji Zhao ^a*^

^a^ *Key Laboratory of High Performance Plastics, Ministry of Education, College of Chemistry, Jilin University, Changchun 130012, PR China.*

* Corresponding author. E-mail: zhaochengji@jlu.edu.cn.

**Contents**

[1 Materials	3](#_Toc231393301)

[2 Methods	3](#_Toc231393302)

[3 Characterizations	5](#_Toc231393303)

[4 Supplementary figures	8](#_Toc231393304)

[5 Supplementary Tables	12](#_Toc231393305)

[6 References	15](#_Toc231393306)

**1 Materials**

Vanillin (VAN, ≥99%), protocatechualdehyde (PCA, ≥99%), potassium carbonate (K₂CO₃, ≥98%), 1,3-bis(3-aminopropyl)tetramethyldisiloxane (BAS, ≥95%), and vanillylamine hydrochloride (≥99%) were purchased from Shanghai Aladdin Biochemical Technology Co., Ltd. Methanol (MeOH, ≥99.5%), epichlorohydrin (ECH, ≥99.5%), cadaverine (CAD, 97%), and tetrabutylammonium bromide (TBAB, ≥99.5%) were purchased from Energy Chemical Co., Ltd., China. Acetone, toluene, tetrahydrofuran (THF), petroleum ether, and N,N-dimethylformamide (DMF) were purchased from Sinopharm Chemical Reagent Co., Ltd., China. Sodium hydroxide (NaOH, 99%), sodium sulfate (Na₂SO₄, 99%), ethyl acetate, and 1,5,7-triazabicyclo[4.4.0]dec-5-ene (TBD, 97%) were purchased from Tianjin Sinochem Biochemical Co., Ltd. All reagents were used as received without further purification. Plain-weave T300 1K carbon fiber fabric was obtained from Toray Industries, Inc., Japan.

**2 Methods**

**2.1 Synthesis of vanillin-based** **epoxy monomer (VAN-EP) and protocatechualdehyde-based epoxy monomer (PCA-EP)**

The synthesis of epoxy monomer was performed by two steps. First, vanillylamine hydrochloride (37.93 g, 0.2 mol) and anhydrous potassium carbonate (27.64 g, 0.2 mol) were dissolved into anhydrous methanol (150 mL) in a 250 mL three-necked flask equipped with a reflux condenser. Subsequently, vanillin (34.4 g, 0.2 mol) was added dropwise under nitrogen at 60 ℃, and the mixture was stirred for 5 h. After cooling to room temperature, the reaction mixture was poured into deionized water (1000 mL) to precipitate the product. The precipitate was collected by vacuum filtration, washed three times with deionized water, and dried under vacuum at 50 ℃ for 12 h, yielding a yellow powder designated as the vanillin-based bisphenol monomer (VAN-AM). The yield of VAN-AM was 87%.

Next, VAN-AM (57.46 g, 0.2 mol), epichlorohydrin (ECH, 160 mL, 2 mol), and tetrabutylammonium bromide (TBAB, 5.7 g, 10 wt% relative to VAN-AM) were charged into a 500 mL three-necked flask equipped with a reflux condenser. The mixture was stirred at 80 ℃ for 3 h under nitrogen. It was then cooled to 5 ℃ in an ice bath, followed by the dropwise addition of 40 wt% aqueous NaOH (32 g, 0.32 mol). The resulting mixture was stirred at room temperature for 5 h. Upon completion, ethyl acetate (50 mL) was added, and the organic phase was washed three times with deionized water. The combined organic phase was dried by anhydrous sodium sulfate overnight. The dried solution was concentrated under reduced pressure at 60 ℃ using a rotary evaporator and further dried in an oven at 60 ℃ for 12 h. The obtained crude product was purified by column chromatography, affording pure VAN-EP as a yellow liquid in 80% yield. Using the similar procedure, the protocatechualdehyde-based epoxy monomer (PCA-EP) was synthesized as a deep-red liquid in 70% yield.

**2.2 Preparation of bio-based epoxy Vitrimers and Carbon Fiber Reinforced Composites**

A catalytic amount of TBD was added to VAN-EP and stirred until fully dissolved at 60 ℃. The mixture was then cooled to 30 ℃, after which the curing agent BAS was added and thoroughly mixed. The mixture was subsequently degassed under vacuum for 30 min and poured into a custom mold. The molar ratio of N-H groups in BAS to epoxy groups in VAN-EP was fixed at 1:1. Curing procedure was performed in three sequential stages: 60 ℃ for 10 h, 100 ℃ for 4 h, and 120 ℃ for 4 h. The resulting epoxy vitrimer was designated VAN-BAS. PCA-BAS was prepared analogously for comparison using PCA-EP as the epoxy monomer. Their carbon fiber-reinforced composites, denoted CF/VAN-BAS and CF/PCA-BAS, were fabricated by the hand lay-up method. The composites were subsequently hot-pressed at 60 ℃, 100 ℃, and 120 ℃ for 4 h under 10 MPa pressure. The carbon fiber fabric content in the composites was approximately 60 wt%.

**3 Characterizations**

The ^1^H NMR spectra were collected through a Bruker-Avance III 400 MHz spectrometer, with CDCl_3_ employed as the solvent.

The Nicolet iS10 was used to obtain the infrared spectra (FT-IR) of the monomers and specimens using the ATR pattern.

The Thermo Scientific Q Exactive Orbitrap of USA was used for high-resolution mass spectrometry (HR-MS) of the bisphenol monomer and epoxy monomer. Test Mode: 1-negative, RT: 0.08-1.08, AV: 99, T: FTMS- p ESI ms [50.0000-750.0000]

The thermal stability was determined by thermogravimetric analyses (TGA) with a Mettler Toledo TGA instrument from 80 ℃ to 600 ℃ at a heating rate of 10 ℃ min^-1^ under a nitrogen flow of 20 mL min^-1^. Before testing, the specimens were dried at 120 ℃ for 2 h to remove moisture and then cooled to ambient temperature.

The non-isothermal DSC study was performed using TA DSC Q20 equipment. The specimens (5 ~ 10 mg) were placed into aluminum crucible and subjected to dynamic DSC analysis in the temperature range from 40 ℃ to 450 ℃ with varying heating rates of 5, 10, 15, 20, and 25 K min^-1^ under N_2_ atmosphere, utilizing an empty crucible as the point of comparison. The Kissinger-Ozawa-Crane methodology was used to determine the reaction activation energy (*E*_a_) and *n*th order for the curing process.

The dynamic mechanical properties of the specimens were carried out by using TA DMA Q850 in single cantilever mode with a preload force of 0.01 N, and a frequency of 1 Hz. Data were recorded from 25 °C to a defined temperature at a heating rate of 3 °C min^-1^.

Empirical Equations for Rubber Elasticity：

$$\rho=\frac{E^{'}}{3RT}$$

(1)

where *E*' is the storage modulus at *T*_g_ +30 ℃, *R* is 8.314 J mol^-1^ K^-1^, *T* refers to the corresponding temperature of *T*_g_ +30 ℃.

Stress relaxation curves were obtained at various temperatures using the TA DMA Q850 instrument. A prestressed of 0.01 N was given to ensure straightness and a 1% deformation was used under the testing.

Arrhenius’ Equation：

$$\tau\left( T \right)=\tau_{0} exp(\frac{E_{a}}{RT})$$

(2)

where R is 8.314 J mol^-1^ K^-1^, T is the experimental temperature, τ is the stress relaxation time.

Dumbbell-shaped samples were subjected to tensile tests using electronic universal testing equipment (SHIMADZU, AG-I) at the speed of 2 mm min^-1^.

The surface morphology was observed by scanning electron microscopy (SEM, Nova nano 450) with an accelerating potential of 10 kV. The surface treatment of the material is done by gold spraying before testing.

The self-repairing property of epoxy vitrimer was investigated by observing the changes of surface cracks through CNOPTEC polarised light microscope. Samples were made into a thin sheet, and scratches were made on the sheet with a razor blade, and the changes in the scratches were observed and measured with the heating time at 200 °C. The repair efficiency was calculated using the following formula,

$\xi=$ $\frac{W_{1}-W_{0}}{W_{0}}\times100\%$

where $W_{1}$ is the width of cracks after heating, and $W_{0}$ is the width of the original cracks.

The tensile properties of CFRP samples with dimensions of 80 mm × 6 mm × 1.12 mm were measured using an Electric Universal Testing Machine (Instron 5567, USA) with a gauge length of 40 mm and a stretching rate of 5 mm⋅min^-1^.

Raman spectra were recorded on a Renishaw RM1000 Raman spectrograph. The laser power measured at the samples was below 10.0 mW for 514 nm radiation. The scanning wavenumber range was 100 cm^-1^ to 3200 cm^-1^.

X-ray diffraction (XRD) measurements were made from the filament of virgin and recovered CFs. The diffraction studies were performed in reflection mode using X-ray diffractometer, Bruker AXS D8 Discover using Cu Ka radiation at a scan rate of 0.2°/s in a 2h of 5–60°, and operated at 30 kV and 20 mA.

X-ray photoelectron spectroscopy (XPS) measurements were conducted on a Thermo Scientific K-Alpha attached to a data acquisition system with a base vacuum of 3.0 × 10^-7^ mbar. Spectra were referenced to the C1s peak at 284.8 eV.

The single fiber tensile property of the recovered CF was tested by SHIMADZU, AG-I. Because the recovered CF was short and disordered, the samples cannot be tested according to the standard method. The strength of CF was determined as follows. Single filament was selected from T300-1k with a length of about 2.5 cm. The distance between the two clamps was 2 cm. The stretching speed was 20 mm/min. At least 10 filaments were tested for each sample.

The in-situ FTIR was conducted on German Bruker VERTEX 80v spectrophotometer (Bruker, Germany) under a N_2_ atmosphere in the temperature range of 25 to 200 °C at 5 °C/min. 25 ℃, 50 ℃, 75 ℃, 100 ℃, 150 ℃ and 200 ℃ were selected for scanning recordings and held at each temperature for 5 minutes.

The internal structure and defect distribution of CFRP was tested by Scanning Acoustic Microscope (SAM 300B), German PVA TEPLA. Inspection with transmitted signal (T-scan). Scanning range: 100 mm×100 mm, ultrasonic transducer: 30MHz, Ultrasonic medium: distilled water.

This study employs the GROMACS software package to conduct molecular dynamics simulations, investigating the spatial distribution of polymer chains within two crosslinked networks: PCA-BAS and VAN-BAS. The Universal Force Field was utilized to describe the polymers, with force field parameters accounting for interactions between units within the polymer chains. First, the system stability was ensured through an energy minimization process, with a maximum force threshold set at 100 kJ/mol·nm [1]. Subsequently, the system underwent 2 ns of temperature equilibration under NVT conditions, employing a V-rescale thermostat to maintain a temperature of 300 K with a coupling time constant of 0.1 ps [2]. Subsequently, an NPT equilibration phase was conducted for 4 ns, maintaining a pressure of 1 bar via the Parrinello–Rahman pressure coupler [3]. Finally, a 40 ns production-stage MD simulation followed the NPT equilibration, employing a time step of 2 fs and the LINCS algorithm to constrain hydrogen bonds within the polymer chains [4]. The simulations focused on analyzing the effect of repeating units on molecular chain stacking within different polymer networks, exploring how varying repeating units influence chain arrangement and spatial distribution.

**4 Supplementary figures**

Figure S1. FTIR spectra of bio-based bisphenol Monomers VAN-AM and PCA-AM.

Figure S2. The High-resolution mass spectrometry of VAN-AM.

Figure S3. The High-resolution mass spectrometry of PCA-AM.

Figure S4. ^1^H-NMR Spectra of Bisphenol Monomers and Epoxy Monomers.

Figure S5. FTIR spectra of VAN-EP, PCA-EP, VAN-BAS, and PCA-BAS.

Figure S6. Tensile strength and tensile modulus of VAN-BAS and PCA-BAS

Figure S7. Flexural strength and tensile modulus of VAN-BAS and PCA-BAS

Figure S8. SEM images of fracture surfaces of VAN-BAS and PCA-BAS after impact tests.

Figure S9. Epoxy resin formed through hot-press molding (using VAN-BAS as an example; the edges of the resin have been trimmed).

Figure S10. Digital photo of the welded CFRP used to lift approximately 3 kg bottled water.

Figure S11. Solvent resistance of a bio-based epoxy vitrimer: VAN-BAS as an example.

Figure S12. The degradation process of the bio-based epoxy resin VNA-BAS involves grinding the resulting particles and reforming them via hot pressing. The reference sample, VAN-CAD, is an epoxy resin specimen cured using the same process but with cadaverine replacing the curing agent.

**5 Supplementary Tables**

Table S1. DMA parameters of bio-based epoxy vitrimers

|  | Eʹ (MPa) | T_g_ (℃) | υ_e_ (mol m^-3^) |
| --- | --- | --- | --- |
| VAN-BAS | 3900 | 116 | 2751 |
| PCA-BAS | 5115 | 127 | 3880 |

Table S2. Mechanical properties of bio-based epoxy vitrimers

|  | Tensile strength (MPa) | Tensile modulus (MPa) | Flexural strength  (MPa) | Flexural modulus  (GPa) | Impact strength  (kJ·m^-2^) | | |
| --- | --- | --- | --- | --- | --- | --- | --- |
|  |  |  |  |  | Notched | Un-notched | |
| VAN-BAS | 94.3 ± 12.4 | 1433.6 ± 79.3 | 168.7 ± 20.4 | 4.2 ± 0.4 | 11.5 ± 0.6 | | 39.1 ± 1.4 |
| PCA-BAS | 74.8 ± 11.6 | 1826.8 ± 90.5 | 128.6 ± 19.8 | 5.3 ± 0.5 | 10.4 ± 0.8 | | 26.7 ± 2.5 |

Table S3. Comparison of T_g_ and tensile strength between VAN-BAS and other reported epoxy vitrimers.

|  | Tensile strength (MPa) | T_g_ (℃) | References |
| --- | --- | --- | --- |
| VAN-BAS | 94.3 | 116 | This work |
| ESO-QC/N-MI 1.4 | 37.2 | 115 | [5] |
| ESO-QC/His 1.4 | 44.2 | 86 |  |
| BVT-5 | 68 | 100 | [6] |
| BVT-0 | 47 | 74.2 |  |
| AAD-EIA | 37 | 45 | [7] |
| BDEF-EP-AFD | 42 | 127 | [8] |
| Eu-EP-3/IPDA | 35 | 63.9 | [9] |
| BVF-EP/AFD-R=0.5 | 26 | 110 | [10] |
| A9P1-D230 | 63.4 | 82.8 | [11] |
| DGEBA/AEESN | 55.4 | 59.8 | [12] |
| DFBPA-EP/CA | 33 | 75 | [13] |
| DFBPA-EP/HA | 39.53 | 106 |  |
| DGEBA/BAS | 79.3 | 137 | This work |
| DGEBA/DDS | 87.9 | 148 | This work |

Table S4. Thermal stability properties of bio-based epoxy vitrimers

|  | T_d5%_, (℃) | T_d10%_, (℃) | T_max_, (℃) | Char yield at 800 ℃, (%) |
| --- | --- | --- | --- | --- |
| VAN-BAS | 294 | 310 | 360 | 27.9 |
| PCA-BAS | 307 | 315 | 369 | 37.1 |

Table S5. Theoretical dynamic parameters of the stress relaxation process

|  | τ^*^ (200 ℃), s | *E*_a_, kJ mol^-1^ | T_v_, ℃ |
| --- | --- | --- | --- |
| VAN-BAS | 45.6 | 59.6 | 49.7 |
| PCA-BAS | 65.2 | 70.5 | 68.3 |

Table S6. Dynamic covalent bond content in bio-based epoxy vitrimers.

|  | VAN-BAS | PCA-BAS |
| --- | --- | --- |
| Epoxy monomer content (g) | 10.0 | 10.0 |
| Curing agent content (g) | 3.11 | 4.22 |
| TBD content (mmol) | 1.25 | 1.70 |
| Imine bond content (mol) | 0.025 | 0.067 |
| Siloxane bond content (mol) | 0.012 | 0.017 |
| Total content of dynamic covalent bonds (mol) | 0.037 | 0.084 |

EEW value of VAN-EP: 0.50 eq/100 g, EEW value of PCA-EP: 0.68 eq/100 g

The N-H/epoxy resin ratio was 1:1; TBD content was 10 mol % of BAS

Table S7. Scratch self-repairing width and efficiency

|  | Initial scratch width (μm) | After 20 min | | After 40 min | | | After 60 min | | |  |
| --- | --- | --- | --- | --- | --- | --- | --- | --- | --- | --- |
|  |  | scratch width (μm) | Repair efficiency | | scratch width (μm) | Repair efficiency | | scratch width (μm) | Repair efficiency | |
| VAN-BAS | 18.45 | 10.68 | 42.1% | | 4.86 | 73.6% | | 0 | 100% | |
| PCA-BAS | 17.48 | 14.57 | 16.6% | | 10.68 | 38.9% | | 3.88 | 77% | |

Table S8. Thermomechanical properties of bio-based epoxy vitrimers after remodeling.

|  | Tensile Testing | | DMA | |
| --- | --- | --- | --- | --- |
|  | Tensile strength (MPa) | Elongation (%) | Eʹ (MPa) | T_g_ (℃) |
| VAN-BAS (R0) | 94.3 | 6.9 | 3900 | 116 |
| R1 | 60.2 | 5.0 | 3879 | 119 |
| R2 | 53.8 | 4.9 | 3862 | 120 |
| PCA-BAS (R0) | 74.8 | 4.6 | 5115 | 127 |
| R1 | 45.2 | 4.2 | 4642 | 125 |
| R2 | 38.5 | 3.7 | 4833 | 126 |

Table S9. Mechanical Properties of Bio-based Composites

|  | Tensile strength (MPa) | Flexural strength (MPa) | Interlayer shear strength (MPa) |
| --- | --- | --- | --- |
| CF/VAN-BAS | 605 ± 36 | 628 ± 49 | 68 ± 3.5 |
| CF/PCA-BAS | 546 ± 38 | 518 ± 53 | 55 ± 2.8 |

Table S10. Comparison tensile strength between CF/VAN-BAS and other reported CFRP.

|  | Tensile strength (MPa) | References |
| --- | --- | --- |
| CF/VAN-BAS | 605 | This work |
| FPA/GTE/HBP15−CF | 585 | [14] |
| CF/BVT-0 | 486 | [15] |
| Zn-DGEBA/TOSMA@CFs (3) | 450 | [16] |
| Gte-VA-CF | 449 | [17] |
| Dual disulfide CFRP | 334 | [18] |
| BDEF-EP-AFD/CF | 223 | [19] |
| CF/HPCA-TAA-EP-0.7 | 188 | [20] |
| A7P3-D230/CF | 187 | [21] |
| ESO-VA-CF | 145 | [22] |
| ETOD-SA-CF | 120 |  |

Table S11. Comparison lap shear strength between CF/VAN-BAS and other reported CFRP.

|  | lap shear strength (MPa) | References |
| --- | --- | --- |
| CF/VAN-BAS | 31.3 | This work |
| CF/PCA-BAS | 27.2 |  |
| vGFRC | 10.8 | [23] |
| vCFRC | 17.6 |  |
| CF-Vitrimax T100 | 12.0 | [24] |
| CFRP | 13.0 | [25] |
| CFRP-Ind250 | 22.4 | [26] |
| CFRP-Us 250 | 27.9 |  |
| CFRP-Us75 | 24.3 |  |

**6 References**

[1]. Hess, B.; et al. Lincs: A Linear Constraint Solver for Molecular Simulations, Journal of Computational Chemistry 1997, 18, 1463–1472.
[2]. Bussi, G.; Donadio, D.; Parrinello, M. Canonical sampling through velocity rescaling, Journal of Physical Chemistry 2007, 126, 014101.
[3]. Parrinello, M.; Rahman, A. Polymorphic transitions in single crystals: A new molecular dynamics method, Journal of Applied Physics 1981, 52, 7182–7190.
[4]. Lindahl, E.; et al. Flexible protein–ligand docking with a molecular dynamics approach, Journal of Computational Chemistry 2001, 22, 205–215.
[5]. X.-M. Ding, L. Chen, D.-M. Guo, B.-W. Liu, X. Luo, Y.-F. Lei, H.-Y. Zhong, Y.-Z. Wang, Controlling Cross-Linking Networks with Different Imidazole Accelerators toward High-Performance Epoxidized Soybean Oil-Based Thermosets, ACS Sustainable Chemistry & Engineering 9(8) (2021) 3267–3277.
[6]. K. Dong, D. Zhao, Y. Pang, C. Zhao, Vanillin-derived multifunctional reinforcing agent enabling recyclable epoxy vitrimer composites via hydrogen/dynamic covalent collaborative networks, Polymer Degradation and Stability 241 (2025) 111608.
[7]. Z. Yu, S. Ma, Z. Tang, Y. Liu, X. Xu, Q. Li, K. Zhang, B. Wang, S. Wang, J. Zhu, Amino acids as latent curing agents and their application in fully bio-based epoxy resins, Green Chemistry 23(17) (2021) 6566–6575.
[8]. K. Dong, D. Zhao, Y. Pang, B. Liu, Q. Liu, T. Mu, C. Zhao, Multiple-reprocessable guaiacol-derived epoxy vitrimer with disulfide crosslinks and closed-loop recycling of carbon fiber-reinforced composites, Chemical Engineering Journal 508 (2025) 160754.
[9]. L. Dai, Y. Zhang, F. Ma, T. Ji, L. Zhang, B. Lyu, Eugenol-Based Epoxy Materials with High Biobased Carbon Content and Their Structure–Property Relationships, ACS Applied Polymer Materials 7(2) (2025) 1062–1073.
[10]. K. Dong, S. Tang, D. Zhao, Y. Pang, C. Zhao, Vanillin-derived bio-based epoxy resins containing dual dynamic Schiff base and disulfide bonds with reprocessability and degradability, Polymer Degradation and Stability 230 (2024) 111077.
[11]. W. Yang, H. Ding, W. Zhou, T. Liu, P. Xu, D. Puglia, J.M. Kenny, P. Ma, Design of inherent fire retarding and degradable bio-based epoxy vitrimer with excellent self-healing and mechanical reprocessability, Composites Science and Technology 230 (2022) 109776.
[12]. Z. Li, X. Ji, X. Li, D. Gao, L. Shen, W. He, J. Sun, D. Lu, J. Meng, K. Guo, Eco-friendly eugenol curing agent-derived biobased epoxy resins for multi-functional integration, Chemical Engineering Journal 522 (2025) 167486.
[13]. S. Chai, Y. Fang, Z. Chen, D. Kong, S. Xiang, S. Zhao, F. Fu, X. Liu, Constructing Fully Biobased Epoxy Vitrimer Based on Disulfide Bonds with Desired Mechanical, Recyclability, and Degradability Properties, ACS Applied Polymer Materials 7(6) (2025) 3981–3990.
[14]. Y. Xu, H. Zhang, S. Dai, S. Xu, J. Wang, L. Bi, J. Jiang, Y. Chen, Hyperbranched polyester catalyzed self-healing bio-based vitrimer for closed-loop recyclable carbon fiber-reinforced polymers, Composites Science and Technology 228 (2022) 109676.
[15]. K. Dong, D. Zhao, Y. Pang, C. Zhao, Vanillin-derived multifunctional reinforcing agent enabling recyclable epoxy vitrimer composites via hydrogen/dynamic covalent collaborative networks, Polymer Degradation and Stability 241 (2025) 111608.
[16]. W. Li, L. Xiao, J. Huang, Y. Wang, X. Nie, J. Chen, Bio-based epoxy vitrimer for recyclable and carbon fiber reinforced materials: Synthesis and structure-property relationship, Composites Science and Technology 227 (2022) 109575.
[17]. Y.-Y. Liu, G.-L. Liu, Y.-D. Li, Y. Weng, J.-B. Zeng, Biobased High-Performance Epoxy Vitrimer with UV Shielding for Recyclable Carbon Fiber Reinforced Composites, ACS Sustainable Chemistry & Engineering 9(12) (2021) 4638–4647.
[18]. H. Si, L. Zhou, Y. Wu, L. Song, M. Kang, X. Zhao, M. Chen, Rapidly reprocessable, degradable epoxy vitrimer and recyclable carbon fiber reinforced thermoset composites relied on high contents of exchangeable aromatic disulfide crosslinks, Composites Part B: Engineering 199 (2020) 108278.
[19]. K. Dong, D. Zhao, Y. Pang, B. Liu, Q. Liu, T. Mu, C. Zhao, Multiple-reprocessable guaiacol-derived epoxy vitrimer with disulfide crosslinks and closed-loop recycling of carbon fiber-reinforced composites, Chemical Engineering Journal 508 (2025) 160754.
[20]. Y. He, Y. Liu, Z. Yu, X. Zhou, J. Zhu, Z. Tang, Dynamic covalent epoxy network of hyperbranched-synergistic-supramolecular: Catalyst-free reprocessing, and application in carbon fiber composites recycling, Supramolecular Materials 3 (2024) 100078.
[21]. W. Yang, H. Ding, W. Zhou, T. Liu, P. Xu, D. Puglia, J.M. Kenny, P. Ma, Design of inherent fire retarding and degradable bio-based epoxy vitrimer with excellent self-healing and mechanical reprocessability, Composites Science and Technology 230 (2022) 109776.
[22]. Y.-Y. Liu, J. He, Y.-D. Li, X.-L. Zhao, J.-B. Zeng, Biobased epoxy vitrimer from epoxidized soybean oil for reprocessable and recyclable carbon fiber reinforced composite, Composites Communications 22 (2020) 100445.
[23]. Agni K. Biswal, Ankush Nandi, Hung Wang, [Aniruddh Vashisth](https://www.sciencedirect.com/author/56071228800/aniruddh-vashisth). Ultrasonic welding of fiber reinforced vitrimer composites, Composites Science and Technology 242 (2023) 110202.
[24]. Martinez, P.; Nutt, S. Resistance Welding of Carbon Fiber Reinforced Vitrimer Composites, Journal of Composites Science 12 (2024) 498.
[25]. Hongzhi Feng, Xiwei Xu, Binbo Wang, Yi Su, Yanlin Liu, Chuanzhi Zhang, [Songqi Ma. Facile preparation, closed-loop recycling of multifunctional carbon fiber reinforced polymer composites, Composites Part B: Engineering 257 (2023) 110677.](https://www.sciencedirect.com/author/35734235600/songqi-ma)

[26]. [Francesca Lionetto](https://www.sciencedirect.com/author/55916795700/francesca-lionetto), MariaNicolas Morillas, Silvio Pappadà, Giuseppe Buccoliero, [Irene Fernandez Villegas](https://www.sciencedirect.com/author/57210343119/irene-fernandez-villegas), [Alfonso Maffezzoli.](https://www.sciencedirect.com/author/7004426713/a-m-maffezzoli) Hybrid welding of carbon-fiber reinforced epoxy based composites, Composites: Part A 104 (2018) 32-40.
